# Exploring the Use of Brain-Computer Interfaces in Stroke Neurorehabilitation

**DOI:** 10.1155/2021/9967348

**Published:** 2021-06-18

**Authors:** Siyu Yang, Ruobing Li, Hongtao Li, Ke Xu, Yuqing Shi, Qingyong Wang, Tiansong Yang, Xiaowei Sun

**Affiliations:** ^1^Heilongjiang University of Chinese Medicine, 24 Heping Road, Xiangfang District, Harbin, China 8615-0040; ^2^First Affiliated Hospital, Heilongjiang University of Chinese Medicine, 26 Heping Road, Xiangfang District, Harbin, China 8615-0040; ^3^Shenzhen People's Hospital, Second Clinical Medical College of Jinan University, Department of Rehabilitation Medicine, Shenzhen 518120, China

## Abstract

With the continuous development of artificial intelligence technology, “brain-computer interfaces” are gradually entering the field of medical rehabilitation. As a result, brain-computer interfaces (BCIs) have been included in many countries' strategic plans for innovating this field, and subsequently, major funding and talent have been invested in this technology. In neurological rehabilitation for stroke patients, the use of BCIs opens up a new chapter in “top-down” rehabilitation. In our study, we first reviewed the latest BCI technologies, then presented recent research advances and landmark findings in BCI-based neurorehabilitation for stroke patients. Neurorehabilitation was focused on the areas of motor, sensory, speech, cognitive, and environmental interactions. Finally, we summarized the shortcomings of BCI use in the field of stroke neurorehabilitation and the prospects for BCI technology development for rehabilitation.

## 1. Introduction

According to WHO clinical criteria, stroke is defined as “a rapidly developing sign of focal (or global) brain dysfunction lasting more than 24 hours (unless interrupted by death), with no apparent nonvascular cause.” Stroke is the world's second leading cause of death and third leading cause of injury and can cause severe cognitive, emotional, and sensorimotor impairment in patients [1]. Most stroke victims survive the initial event, and the greatest impact of stroke disease is usually the long-term effect it has on the patient and their family [2, 3]. Unfortunately, there are significant gaps between countries in the quality of stroke research and the effectiveness of medical interventions [4]. Over the last decade, advances in the medical treatment of stroke patients have resulted in a substantial reduction in mortality rates. However, one-third of the 16 million patients worldwide remain disabled each year [5].

In traditional rehabilitation, the gold standard in care for poststroke recovery is a combination of specialized training and general aerobic exercise. Bimanual arm training (BAT) and constraint-induced movement therapy (CIMT) are two of the most established methods for treating stroke-related sports injuries [6]. These rehabilitation techniques are bottom-up interventions that focus on distal limb modulation to cause subsequent improvements in the neural circuits involved in motor recovery. However, even with intensive task-specific training and physical activity, 15-30% of people who have had a stroke are permanently disabled. As a result, many bottom-up interventions are ineffective in stroke patients who have very limited upper limb mobility (Fugl-Meyer score 20) [7]. We need to explore and develop more effective stroke rehabilitation strategies that supplement or replace traditional rehabilitation training.

The remodeling of neurological function after stroke may facilitate the development of new interventions for poststroke rehabilitation, and recent therapeutic options have shifted to facilitating neural circuit reorganization in order to restore motor function. These top-down approaches to rehabilitation are largely due to the mechanisms of brain plasticity [8]. The advancement of artificial intelligence methods and a better understanding of brain plasticity are also critical for functional movement recovery. The human brain's ability to adapt to change and environmental stimuli (brain injury, treatment, and experience) by reorganizing its structure, function, and connections is known as brain plasticity [9]. The basic structural reserve and anatomical plasticity of the brain are important parameters for significant motor recovery [10]. Therefore, the key challenge is to figure out how to optimize neuroplasticity during treatment while also reinforcing connections across the infarcted region and promote creation of new connections, thus facilitating long-term functional recovery.

With the advancement of science and technology, artificial intelligence technologies, such as brain-computer interface (BCI), virtual reality (VR), and augmented reality (AR), are rapidly developing and are gradually being applied in the field of medicine. Due to its direct action on the brain, BCI induces brain plasticity and promotes functional reorganization of the brain, proving to be a superior approach in poststroke rehabilitation, especially for improving motor function in stroke patients. The limited neurorehabilitation modalities are no longer adequate to meet increasing rehabilitation needs of patients with central injuries, and BCI has been shown to be effective in improving motor function and enhancing the lives of stroke patients. In this review, we first examined the latest BCI technologies, including how BCIs are acquired, how signals are processed, and how other artificial intelligence technologies are combined with BCIs, such as functional electrical stimulation (FES) technology, virtual reality, exoskeletons, orthotics, and intelligent wheelchairs. We then presented the specific applications, mechanisms of action, and efficacy of BCI in the treatment of poststroke neural remodeling, such as in BCI-based neurorehabilitation of stroke patients in motor, sensory, verbal, cognitive, and environmental interactions. Finally, we summarized our recent research findings and shortcomings, as well as an outlook on the development of BCI technology in the field of rehabilitation.

## 2. BCI Technology

**Figure 1:**
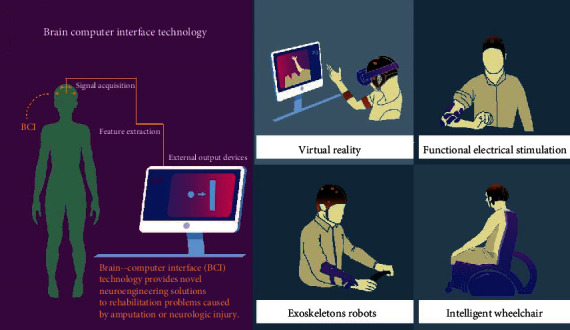
Brain-computer interface for the acquisition, extraction, and conversion of signals from the brain for the ultimate application of controlling external devices: virtual reality, functional electrical stimulation, exoskeleton robots, and intelligent wheelchairs.

The word “brain-computer interface” was first formally identified as “a communication device that does not depend on the usual output pathways of the peripheral nerves and muscles of the brain” at the First International Conference on Brain-Computer Interface Technology in June 1999 [11]. The brain-computer interface (BCI) is a new technology that enables interaction with one's environment through brain signals. This technology takes physiological measurements of mental states directly from the brain and converts them into control signals that can be used to control external devices or computers [12]. The BCI recognizes a set of patterns in brain signals by going through four successive stages: signal acquisition, feature extraction, feature transformation, and device output [13] (Figure 1).

### 2.1. Signal Acquisition Techniques

A BCI uses signals from the brain to gather information about the user's intentions. To do this, the BCI relies on a recording phase to measure brain activity and will then convert that information into an electrical signal that can be easily processed. Depending on the BCI's level of invasiveness, there are two types of recording methods: invasive and noninvasive. Invasive recording methods have more spatial and temporal precision, but they also come with the dangers that come with surgically implantable instruments. Extensive research into noninvasive recording techniques has quickly increased due to the technique's noninvasive and safe nature. Due to the low quality of collected signals and susceptibility to interference, enhancing the signal quality of noninvasive brain-computer interfaces has become a focus of research. Depending on the form of signal acquisition, noninvasive BCIs are divided into electroencephalography (EEG), electrocorticography (ECoG), magnetoencephalography (MEG), intracortical electrical signal mapping (INR), near-infrared spectroscopy (NIRS), functional magnetic resonance imaging (fMRI), and many more. The classification of these acquired signals relies on two types of brain activity: (I) electrophysiological and (II) hemodynamic. These neural signal acquisition methods differ mainly in terms of activity detection, temporal and spatial resolution, safety, and mobility [14] (Table 1).

### 2.2. Feature Extraction

The BCI operation's signal processing phase is split into two sections. Feature extraction is the first step, which extracts the signal features that encode the user's purpose. Different types of thought generate different patterns of brain signals, and the BCI classifies each pattern into a category based on their characteristics. Depending on the type of control signals in the BCI, the patterns can be divided into visual evoked potentials (VEPs), slow cortical potentials (SCPs), P300 evoked potentials (P300), or sensorimotor rhythms (*μ* and *β* rhythms). In this step, the digitized data is algorithmically filtered to remove confounding artifacts, such as 60 Hz noise or EMG activity, to ensure accurate measurements of brain signal characteristics [13].

### 2.3. Feature Translation

The second step in signal processing uses a conversion algorithm that converts extracted signal features into system commands. Brain electrophysiological features or parameters are converted into commands that will generate outputs, such as letter selection, cursor movement, and regulation of a motorized prosthetic, and influence additional assistive devices. The conversion algorithm must be dynamic to accommodate continuous changes in signal characteristics and ensure that the range of the user's specific signal characteristics fully covers the range of control for the device [14].

### 2.4. External Output Devices

End effectors are external output devices that are operated by BCI commands. The function and design of these devices vary depending on the intent of the BCI system and the needs of each end user. Here, we will focus on motor control and neurorehabilitation as end effector targets for BCIs, including functional electrical stimulation (FES), VR, intelligent wheelchairs, orthotics, and exoskeletal robotic devices.

#### 2.4.1. BCI-FES

Functional electrical stimulation (FES) technology works by sending electrical impulses to a paralyzed or damaged limb in order to produce artificial muscle contractions [15]. The benefits of BCI-FES therapy include the ability to promote functional recovery and purposeful plasticity by activating the body's natural efferent and afferent pathways, thereby facilitating motor learning and neural reorganization [16]. One study showed the additional psychological advantages of rehabilitation using BCI-FES with patients, such as increased self-esteem and reduced depression [17]. Another study showed that BCI-FES-treated stroke patients performed skilled and coordinated grasping, made clinically significant progress on tests of upper limb function, and showed improved shoulder subluxation [18]. A new technology, sensory-motor rhythm (SMR) based BCI-FES system, combines the benefits of both technologies, allowing patients with severe disabilities to regain motor function by converting random motor imagery (MI) into physical movements [19]. An enhanced MI-BCI system based on FES and VR has been proposed and validated, unveiling a new era of intelligent rehabilitation therapy [20].

#### 2.4.2. BCI-VR

In recent years, BCI combined with VR technology has become a new technique that has significant applications in neurorehabilitation. Compared to traditional rehabilitation methods, BCI-VR systems can improve individual motivation by increasing the appeal of training, thus shortening the training cycle, providing more effective feedback, and facilitating recovery of brain function [21]. Badia et al. showed that the use of BCI-VR systems could monitor and facilitate cortical reorganization through motor training [22]. Vourvopoulos et al.'s research has shown that a BCI-VR system based on the Reinvent platform can be extremely helpful for patients with severe movement disorders and that the system can be used repeatedly by patients undergoing stroke treatment [23]. A study by Laver et al. also suggests that VR may be beneficial when used as an adjunct to daily care to increase overall treatment time and in turn improve upper limb function and patient autonomy [24]. However, the combination of BCI and VR is only in its infancy, and the current information transfer rates of BCI systems are not ideal. There still remains a long way to go before this technology can be effectively applied to the rehabilitation of patients with neurological diseases.

#### 2.4.3. Orthotics, Exoskeletons, Robotics, and Intelligent Wheelchairs

The main considerations of exoskeletal and orthotic devices for stroke patients are rehabilitation and replacement. An orthotic supports a joint as it moves from static to functional position and can also generate dynamic movements through the range of motion of the joint. This method is useful for patients with low motor neuronal disease or severe muscle atrophy. Ramos-Murguialday et al. developed a new combined EEG-EMG-based BCI technology for use in a BCI-operated hand orthotic neurorehabilitation system, and this technique has thoroughly demonstrated superiority over traditional cortical muscle coherence-based BCI classifications [25]. An et al. have made considerable progress in the application of the BCI-Rogo system to gait orthotics; the combination of behavioral activation of the supraspinal gait region by BCI with the central spinal gait pattern generator via Rogo feedback drive may provide a unique form of Hebbian learning [26, 27]. Rohm et al. developed a hybrid system, which consisted of the FES system and a semiactive orthotic, which restores hand, finger, and elbow function in tetraplegic patients; sustained training with this system can also reverse severe wasting atrophy of paralyzed muscles years after the initial spinal cord injury [28].

Robotic exoskeletons offer the advantages of increased joint strength and reduced load-bearing. Exoskeletons allow soldiers to lift heavy objects and can assist firefighters who have to wear heavy equipment. At the same time, exoskeletons can be utilized to assist the elderly or people with motor impairments in their daily activities. Combining these advantages with BCI technology, the exoskeleton-BCI system can enhance rehabilitation by providing the ability to repeat training exercises to increase the intensity of movement [29]. Bundy et al. demonstrated the effectiveness of a home-used BCI-controlled exoskeleton on motor function in chronic stroke survivors and found a potential correlation between the unaffected cerebral hemisphere and functional recovery [30]. For people with paralysis, restoring unrestrained mobility is another important issue that needs to be addressed. As a result, BCI-driven wheelchairs have become a quickly growing area of research and development. For example, Bundy et al. developed an EEG-based electric wheelchair that detects directional commands via EEG then uses them to directly control the wheelchair. However, more precise handling is more demanding on the user [30].

There is a desire to harness the potential of BCIs to transform the recovery process from neurological disease. Although it is still too early to apply the brain-computer end effector interface in a clinical rehabilitation setting, there has been significant progress in the integration process. Next, we will elaborate on BCI technology and its application in neurorehabilitation after stroke.

## 3. BCI Applications in Nerve Rehabilitation after Stroke

The use of BCI in stroke neurological rehabilitation is a new attempt in modern rehabilitation. Among them, the functionality of BCI in the remodeling of stroke patients' central nervous systems is a pressing topic of inquiry. Neuroplasticity refers to the process by which the brain learns new behaviors, adapts to the environment, and modifies behavior by adding or changing existing synapses. The Hebbian theory, developed by Canadian cognitive psychologist Donald Hebb (1904-1985), suggests that repetitive stimulation of postsynaptic neurons by presynaptic neurons enhances the efficacy of synaptic transmission [31]. Therefore, the aiding technology must match the patient's motor intentions in order to work optimally. In contrast to conventional rehabilitation tools, BCIs can use analysis by exogenous output devices to transform electrical signals from the brain into corresponding commands, thus enabling interaction between the brain and external environment [32]. The brain-limb linkage of hemiplegic patients has greatly increased the motivation of patients, changing from passive acceptance of conventional rehabilitation to active participation in training, which improves the effectiveness of rehabilitation. The replacement and regeneration of impaired neurological function are two essential functions of BCI in recovery [8]. As BCI systems are used to replace missing neurological functions, the user's ability to communicate with and monitor a range of environments and behaviors is restored, including computer-based tasks (word processing, Internet searching, and so on), accessibility devices (powered wheelchair drives) [33], neuroprosthetics [34, 35], and orthotics [25–28]. By inducing activity-dependent brain plasticity, BCI can be used in combination with recovery therapy to help restore normal central nervous system function (Table 2).

### 3.1. Motor Rehabilitation

Long-term motor disability as a result of stroke is one of the main targets of rehabilitation [36]. Even with traditional rehabilitation methods, over two-thirds of survivors develop mild to severe paralysis of the upper and/or lower limbs [3, 37]. It is estimated that nearly 1% of the world's population lives with the sequelae of cerebrovascular events [38]. Impaired motor control [39], general cognitive deficits [40–42], difficulties with speech production or processing [43], and altered mood states [44] are common debilitating effects of stroke [45]. For patients with poststroke motor dysfunction, rehabilitation interventions fall into two categories. The first type is the direct input of externally generated stimuli into the brain (transcranial direct current stimulation, transcranial magnetic stimulation, etc.), and the second type is training for the peripheral limbs. For these purposes, innovative technology-based solutions such as robot-assisted therapy, virtual reality, FES, noninvasive brain stimulation (NIB), and BCI have been suggested [46].

The restoration of upper limb motor defects in serious stroke patients was the initial impetus for the investigation of BCI technology in the field of poststroke rehabilitation. Patients with severe injuries do not have the minimum motor capacity required to undergo traditional rehabilitation therapies, such as occupational therapy (OT) or constraint-induced movement therapy (CIMT), which necessitates the search for a new kind of rehabilitation intervention [45]. The discovery that imagining movement (MI) causes the recruitment of the same neuronal circuits as real movement suggests that BCIs may be useful in recovery. The primary motor cortex (M1) and other brain structures involved in planning and regulating voluntary activity have been shown to be activated by motor imagery [47–50]. For example, studies have shown that motor imagery of clenched fists lowers the threshold of excitation for motor evoked potentials (MEPs) induced by transcranial magnetic stimulation (TMS) delivered to M1 [49]. Numerous studies have shown that BCI treatment can trigger long-term neurological changes and improve upper limb motor function in patients with subacute and chronic strokes [16, 51–57]. Ang et al. published the first report on observed clinical improvement, demonstrating that a BCI-supported robotic rehabilitation system based on motor imagery can improve upper limb motor function in stroke patients and facilitate rehabilitation of the affected hand and wrist in poststroke patients [53]. Eight of the chronic stroke patients had their upper limbs rehabilitated using a BCI. By pushing the damaged hand outwards with the MIT-Manus robot, the BCI device successfully detected MI from real-time EEG signals, based on the MI protocol. On the Fugl-Meyer Assessment Scale, major changes in motor control were reported after 12 recovery sessions over a four-week span (FMA). In 2003, Pfurtscheller et al. [58] were the first to use BCI in combination with FES to enable a tetraplegic patient to grasp a cylinder with his paralyzed hand. Caria et al. showed significant improvement in arm and finger motor function after four weeks of magnetoencephalography-based BCI combined with finger flexion and extension bracing, as well as improvement in a patient in a chronic phase of stroke with severe hand paralysis, using only four weeks of EEG-BCI-robotic arm training [59, 60]. Daly et al. [61] reported in a case of a patient that, after three weeks of BCI-FES training, the affected hand went from having no individual finger separation and no finger extension ability to achieving some degree of independent extension of the index finger.

Taylor et al. performed BCI-based interventions on healthy individuals and stroke patients and recorded motor-related cortical potentials by EEG during MI and ankle dorsiflexion in these subjects, demonstrating that BCI training can affect motor cortical excitability in the lower limbs of both healthy adults and stroke patients [62]. Chung et al. [63] utilized BCI combined with FES for ankle dorsiflexion in stroke patients (observation group) and only FES for ankle dorsiflexion in the control group. After five days of continuous treatment, there was a significant improvement in the standing and walking time test, as well as gait and stride length, in the observation group, while there was no significant improvement in the control group. BCI-based FES training is therefore considered to be more effective than FES alone in improving balance and gait function in stroke patients. Available evidence suggests that BCI training has a significant and immediate effect on improving limb motor function. However, the limited number of studies available does not provide evidence regarding its long-term impact. A large number of clinical trials and the development of new systems must be the focus of the future of motor rehabilitation [64] (Table 3).

### 3.2. Sensory Rehabilitation

While BCIs have made great strides in motor control, significantly less attention has been paid to restoring tactile or skin sensation [65]; haptics is essential for many aspects of motion control [66]. When performing tasks that involve dexterity, such as lighting a match or finding a key, sensory input becomes even more important [65]. Our brains do not separate sensory and motor functions; instead, they construct complex motor strategies and equate desired results to sensory input to make necessary changes. These BCIs will need to combine motor and sensory modalities in order to truly restore function to the arm and hand. Despite the fact that sensory and motor cortices are located in separate anatomical areas, they share the same somatic organization and a critical functional relationship. It should also be remembered that complex and smooth limb movements depend heavily on the incorporation of sensory data to allow for the dynamic adjustment of multiple movement states at the same time [67]. Without tactile signals, our dexterity in grasping and manipulating objects would be severely impaired [68, 69]. Reduced sensory input makes tasks like ascending stairs and walking on uneven ground with a prosthesis challenging, if not risky, for lower limb amputees [69]. Studies on nonhuman primates have shown that the sensory stimulation of closed loops improves motor BCI performance [70]. A promising, recently developed technology now exists that can restore sensorimotor function using a robotic arm controlled by a BCI [66]. However, there is currently no technology available to restore motor function and tactile sensation using the participant's own hands [71–73]. The establishment of bidirectional BCIs on “closed-loop systems” is therefore a new direction for future research to explore.

### 3.3. Communication Rehabilitation

Speech production and communication comprehension deficits afflict up to 30% of people who have had a stroke [74]. Today, the development of BCI technology may be beneficial for the rehabilitation of patients diagnosed with aphasia [75]. Brain-computer interface technology can not only provide a tool for communication but also support neuronal plasticity by activating language circuits, thereby facilitating recovery from aphasia. Three EEG signals for common language communication have been developed alongside EEG-based BCIs and studied: slow cortical potential (SCP), sensorimotor rhythms (SMR), and P300 evoked potentials (P300) [76]. Chaudhary et al. demonstrated for the first time the feasibility of SCP-BCI communication with two locked-in state (LIS) patients diagnosed with amyotrophic lateral sclerosis (ALS) [76]. Lazarou et al. showed that after six months of training, the participant successfully self-regulated his SCP by producing two different brain responses. In the end, he managed to write 454 words in German [77]. Sellers et al. made good progress using noninvasive P300-BCI to rehabilitate communication in LIS patients as well [78]. Kleih et al. applied P300-BCI to five participants diagnosed with poststroke aphasia for communication rehabilitation; participants successfully learned to communicate using a speller with an accuracy rate of 100% [75].

### 3.4. Cognitive Rehabilitation

People who have had a stroke also often suffer from cognitive impairment. Cognitive impairment can be seen as a range of deficits, including poor concentration, slowed information processing, memory impairment, reduced semantic fluency, difficulty producing or processing speech, and aphasia [45]. Most treatments, including motor rehabilitation based on brain-machine interfaces, require a certain minimum level of cognitive ability for the patient to be able to understand and respond to instructions for implementing the rehabilitation program [53, 79, 80]. Patients with severe cognitive impairment who are not capable of meeting these cognitive requirements are automatically excluded from rehabilitation, resulting in a significant reduction in quality of life. Therefore, making most poststroke rehabilitation programs accessible to all patients may be an important facet of cognitive training to consider. Cognitive rehabilitation is defined as a systematic, functionally oriented service of therapeutic activities. The assessment and understanding of the patient's brain behavioral deficits are assessed across many cognitive domains: attention, concentration, memory, comprehension, reasoning, problem solving, judgement, planning, self-monitoring, awareness, and more [81]. Surprisingly, despite the fact that motor function has received a lot of attention in BCI-based rehabilitation and BCIs have shown a lot of promise in promoting motor rehabilitation, there is still a lot of research on using BCIs for poststroke cognitive training [82, 83].

The effects of neurofeedback training based on BCIs have been shown to improve certain cognitive functions in neurodevelopmental and neurodegenerative disorders, such as attention-related hyperactivity disorder (ADHD) [84] and mild cognitive impairment (MCI) in the elderly [85]. Thus, spatially directed enhancement of self-regulation in cortical areas may be another method that can be used for cognitive training. A recent meta-analysis also showed encouraging evidence from several studies demonstrating the effectiveness of neurofeedback-based cognitive training [86]. Gomez-Pilar et al. developed a neurofeedback training (NFT) tool for BCIs based on motor imagery. After NFT training, three cognitive features, visual perception, articulate expression, and immediate memory, all improved dramatically [81, 87]. Martin et al. developed an online cognitive rehabilitation application based on the P300-BCI system for the remote treatment of patients with traumatic brain injury, in conjunction with therapists who will use the BCI at home. This allows therapists to remotely prescribe activities of varying difficulty and offers hope for recovery for people with severe physical and cognitive impairments after a stroke [81]. Furthermore, changes in cognitive outcomes after a stroke can have a concomitant impact on motor function and recovery outcomes. There is a correlation between cognitive and motor function and recovery outcomes, according to several studies [88].

### 3.5. Environmental Interactions

In the technical development of BCI-smart home control systems, the majority only consider younger healthier target populations. However, elderly people with disabilities or limited mobility are more interested in or in higher need of smart homes because of their limited capabilities; being able to operate household appliances at home on their own would greatly enhance their quality of life [89]. BCIs can be used in many different fields: medical applications to control wheelchairs and prostheses [12] or enable people with disabilities to communicate and write texts [90] and general public applications to control toys, video games, or general computer applications. Kosmyna et al. conducted a study using BCIs for the control of smart homes and found that healthy subjects achieved a 77% accuracy on the task given; however, better accuracy was obtained by subjects with disabilities (81%). It was concluded that disabled end-users are more motivated to learn to use the BCI correctly and that the use of BCIs for smart home control is feasible, but further research is needed [89]. Tang et al. developed a brain-driven intelligent wheelchair system that was tested in three patients (cerebral infarction, spinal cord injury, and stroke) and four healthy subjects. Tasks required the user to drive the system close to a walking person and talk to them, to pass through a door into another room, and to pick up a bottle of water on a table and drink it. The results show that the system operates with flexibility and efficiency, with users only needing to issue small commands to receive attentive service. Additionally, it shows that the system is important in accelerating the use of BCIs in real-world settings, particularly for patients using them during rehabilitation [91]. Current wheelchairs, exoskeleton technology, and other smart home developments must continue to be designed to support older or disabled people so that they can continue their daily lives, while also supplementing rehabilitation of deteriorating muscle and motor functions. Smart home environments can ultimately help older people to live independently and feel safe in their own homes.

## 4. Discussion

With the rapid development of modern technology, brain-computer interface (BCI) technology has become an extremely relevant topic in research, and the application of BCIs in the medical field has become one of the more important reasons encouraging its development. While this novel treatment for neurological rehabilitation after stroke is proving to be extremely beneficial, there are a number of areas that need improvement in the field of BCI research. The first area to address is the development of bidirectional BCIs. While we have currently made great progress in BCI motor control, we are at a distinct disadvantage for the recovery of tactile or cutaneous sensation, where the recovery of a limb requires the integration of both motor and sensory modalities. This is where a combination of a bidirectional BCI and a “closed-loop system” that integrates both motor output and sensory input is appealing, where the motor output is adjusted based on the sensory input to achieve the optimal motor route. A neuroprosthetic based on a bidirectional BCI is already under development, and it is just a matter of time until it is applied to stroke neurorehabilitation to better guide clinical practice. The second area that needs to be examined is the application of synchronous versus asynchronous BCI. The stroke BCI rehabilitation systems introduced in this paper, robotic arm, VR, and intelligent wheelchairs, are all synchronous BCI. A synchronous BCI system requires the user to set the EEG data acquisition experimental paradigm to obtain time-specific, real-time acquisition of data; therefore, the user is always “working” in sync with the system. In practice, however, users cannot be in a “working” state for long periods of time, and in most cases, they are usually in a “free” state. In order to improve the usability of online systems and avoid various errors, it is necessary to identify this idle state; to address this need, asynchronous BCIs have been created. There are studies on asynchronous BCI systems, but few of these regard applications to clinical rehabilitation. Asynchronous BCI movements would truly give patients full autonomy in their rehabilitation. Another exciting future possibility is the hybrid brain-machine interface, which requires the use of EEG as well as other physiological signals, such as neuromodulation (noninvasive brain stimulation), electromyographic activity, and heart rate, as input. These combinations can work synergistically to make the control algorithm more robust and to improve the reliability of the user's intent to detect. For example, the existing BCI-robotic arm rehabilitation system only relies on EEG to drive the paralyzed limb in rehabilitation, and there is no active movement of the limb at all. However, under hybrid BCI control, we can use both EEG and EMG to jointly control the manipulator's arm and enhance the “shared control” of the effector's devices. Shared control between the preprogrammed control of the end effector device and the neural control of the human brain-computer interface has potential to improve the performance of motor tasks. Another phenomenon that must be explored is how the quality of signal acquisition of the BCI system directly determines the degree of execution of the effector. In terms of the current classification of signal acquisition methods, there are two types: invasive and noninvasive. Invasive BCI has the advantage of good signal quality, but the disadvantages are that it is very invasive and degrades over time due to damage to the electrode sheet. We therefore need to develop harmless, more stable electrode materials. The advantages of noninvasive BCI are its safety and convenience, but the disadvantage is its poor signal quality. We need to balance the advantages and disadvantages of both devices to find a more efficient way of collecting signals and to continue developing intelligent adaptive neural interfaces.

## 5. Conclusion

This review describes several BCI applications (e.g., motor, sensory, verbal, cognitive, and environmental interactions) to aid in the rehabilitation of stroke patients. We hope that the techniques presented in this paper will further contribute to the design of new applications and devices for BCI-based stroke rehabilitation. Brain-computer interface technology has already demonstrated exciting results in providing cognitive and physical support and rehabilitation, and we look forward to future innovations in this important area of research that will ultimately affect us all.

## Figures and Tables

**Table 1:** Comparison of BCI pivot signal acquisition methods and their advantages and disadvantages.

Nerve-signal acquisition methods	Event monitoring	Time resolution	Spatial resolution	Safety	Advantage	Disadvantage
Electroencephalogram (EEG)	Electrical signals	~0.05 s	~10 mm	Noninvasive	High temporal resolution, relatively low cost, high portability, low risk to users	Poor signal quality
Magnetoencephalography	Magnetic signals	~0.05 s	~5 mm	Noninvasive	High temporal and spatial resolution, less training time, and more reliable communication	Technology is too large and expensive
Electrocorticography	Electrical signals	~0.003 s	~1 mm ~0.5 mm (LEP)	Invasive	High temporal and spatial resolution and low artefact vulnerability	Electrode mesh implanted in craniotomy, harmful to health
Intracortical point signal acquisition	Electrical signals	~0.003 s	~0.1 mm (MUA) ~0.05 mm (SUA)	Invasive	High spatial and temporal resolution	Signal quality and sensitivity diminish with time
Functional MRI	Metabolism	~1 s	~1 mm	Noninvasive	High spatial resolution	Very low time resolution, too large to carry
Near-infrared spectroscopy	Metabolism	~1 s	~5 mm	Noninvasive	Low cost, high portability, and acceptable time resolution on the order of 100 milliseconds	Very low spatial resolution

**Table 2:** Current state of the application of BCIs in the field of stroke rehabilitation.

Current state of the development of BCIs in the field of stroke rehabilitation	
Motor rehabilitation	The use of BCIs is rapidly developing in the field of locomotion, and BCIs are effective in restoring upper and lower extremity motor when used in conjunction with FES, robotics, and robotic arms.
Sensory rehabilitation	Related research is working on sensory-motor modalities for BCIs, and the development of sensory-motor closed-loop systems will improve the efficiency of rehabilitation. However, the development of sensory rehabilitation is still in its initial stage and has not yet been put into clinical use.
Communication rehabilitation	BCIs can not only help restore the rehabilitation of language disorders in stroke but also serve as a substitute for language to restore the ability to communicate in patients with language loss. Currently, the study is based on three main signals: SCP-BCI, SMR-BCI, and P300-BCI.
Cognitive rehabilitation	Applying BCIs to cognitive training improves certain cognitive functions in neurodevelopmental and neurodegenerative diseases, but there are relatively few clinical studies.
Environment interaction	The application of BCIs in environmental interaction is the most humane consideration for the quality of life of stroke patients with hemiplegia. The development of smart homes is greatly increasing interactions between patients and the outside environment.

**Table 3:** Sports rehabilitation author statistics.

Author	BCI facility	Site of action	Result
Ang	BCI-robotic	Upper limb	Improvement
Pfurtscheller	BCI-FES	Hand	Improvement
Caria	BCI-robotic	Arm and finger	Improvement
Daly	BCI-FES	Hand and finger	Improvement
Taylor	BCI-orthotics	Lower limbs	Improvement
Chung	BCI-FES	Ankle	Improvement

## Data Availability

This article is a review article and does not contain relevant data.
